# 613. Clinical Outcomes Following Dalbavancin Administration during Outpatient Parenteral Antimicrobial Therapy

**DOI:** 10.1093/ofid/ofab466.811

**Published:** 2021-12-04

**Authors:** Jessica Tuan, Jehanzeb Kayani, Ann Fisher, Brian Kotansky, Louise Dembry, Louise Dembry, Rupak Datta

**Affiliations:** 1 Yale University School of Medicine, New Haven, CT; 2 Yale University School of Medicine, VA Connecticut Healthcare System, West Haven, CT; 3 VA Connecticut Healthcare System, West Haven, CT; 4 Yale University, New Haven, CT; 5 Yale School of Medicine - Yale New Haven Hospital, West Haven, CT

## Abstract

**Background:**

Dalbavancin, a lipoglycopeptide with prolonged half-life targeting Gram-positive organisms, is approved for treatment of acute bacterial skin and soft tissue infection. It reduces hospital duration in patients with barriers to short-term rehabilitation or outpatient parenteral antimicrobial therapy (OPAT). Increasing evidence supports the off-label use of dalbavancin to treat other types of infection. We conducted a quality improvement study to evaluate outcomes following dalbavancin administration.

**Methods:**

We performed a cohort study of recipients of ≥1 dose of dalbavancin from 1/31/2016-1/31/2021 at the Veterans Affairs Connecticut Healthcare System. Demographic, comorbidity, microbiological, antibiotic duration prior to dalbavancin, indication for dalbavancin, and type of infection data were collected. Outcomes included 1) lab abnormalities: hepatotoxicity within 2 weeks of dalbavancin; 2) clinical cure: resolution of symptoms of infection within 90 days; 3) all-cause readmission within 90 days; and 4) all-cause mortality within 90 days.

**Results:**

42 patients met criteria. Median age was 69 years (range, 32-91), 100% were male, 55% (n=23) had diabetes, 31% (n=13) had liver disease, 36% (n=15) had other immunosuppressive conditions, and 12% (n=5) had substance use disorder (SUD). All received their first dose as inpatients. Median hospital duration was 8 days (range, 1-32). 4 (10%) required critical care. Median antibiotic duration prior to dalbavancin was 7 days (range, 1-42). Indications included ineligibility for OPAT (n=21, 50%), pharmacologic reasons (n=10, 24%), ineligibility for peripherally inserted central catheter (n=6, 14%), or SUD (n=5, 12%). Common microorganisms were *Staphylococcus* spp. (n=22, 52%), polymicrobial (n=13, 31%), and *Corynebacterium* spp. (n=10, 24%). 93% (n=39) had clinical cure of infection; readmissions and mortality were rare (Table 1).

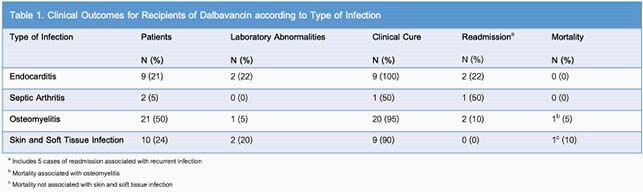

**Conclusion:**

Dalbavancin was associated with clinical cure for diverse infections with low rates of adverse events, readmission and mortality in patients ineligible for traditional OPAT. Although confirmatory data are needed from larger studies, dalbavancin appears to be a versatile therapeutic agent for Gram-positive infections.

**Disclosures:**

**All Authors**: No reported disclosures

